# A Case of Macroenzyme Aspartate Aminotransferase Mimicking Hepatic Injury

**DOI:** 10.7759/cureus.17311

**Published:** 2021-08-19

**Authors:** Sarah Schimming, Jash Bansal, Farhad Sahebjam

**Affiliations:** 1 Department of Internal Medicine, Carilion Clinic, Roanoke, USA; 2 Department of Gastroenterology, Carilion Clinic, Roanoke, USA

**Keywords:** macroenzyme, ast (aspartate aminotransferase), polyethylene glycol, immunoglobulin, transaminase

## Abstract

Macroenzymes are high molecular weight complexes that are formed by the binding of normal serum enzymes with circulating immunoglobulins - primarily IgG, IgA, and IgM. These high molecular weight complexes are difficult to clear through the kidneys, therefore they remain persistently elevated in the serum leading to inaccurate diagnoses and unnecessary workup. The prevalence of macroenzymes is relatively rare in the general population; however, it is an important phenomenon to be aware of when working up isolated elevations in serum enzymes. A quick and efficient test for the detection of macroenzymes is the polyethylene glycol precipitation test. Here, we present a case of macro-aspartate aminotransferase masquerading as an underlying hepatobiliary disease ultimately leading to an extensive evaluation before arriving at the correct diagnosis. This case highlights the importance of an accurate and efficient diagnosis of macroenzymes in the serum in order to prevent unnecessary healthcare utilization and also to decrease the psychological burden on the patient.

## Introduction

Macroenzymes are formed by the aggregation of monomeric immunoglobulins with circulating enzymes, ultimately forming large multimers that are difficult to clear in the kidneys [1-6]. There are two different forms of macroenzymes, macroenzyme type I and macroenzyme type II. The incidence of macroenzymes is relatively rare with a reported prevalence of 0.5-2.5% in the general population [2]. The early detection of macroenzymes is clinically important as it can prevent the unnecessary utilization of healthcare resources and reduce the financial burden on the patient. A simple and economic means for detecting macroenzymes is through the use of polyethylene glycol precipitation testing [4,5]. This case highlights the importance of an accurate diagnosis in the evaluation of isolated aspartate aminotransferase levels.

## Case presentation

A 55-year-old female with a medical history relevant for gastroesophageal reflux disease, obstructive sleep apnea, obesity, asthma, and depression presented as a referral to her gastroenterologist with complaints of vague abdominal pain and persistently elevated aspartate aminotransferase (AST). She had been following with her primary care doctor for symptoms of right-sided abdominal pain and unexpected elevation in her AST for several years. Her primary care doctor obtained a CT of the abdomen and pelvis, abdominal ultrasound, and initial laboratory tests, including a complete blood count (CBC) and basic metabolic panel (BMP), all of which were within normal limits. She was referred to a gastroenterologist for further workup.

**Figure 1:**
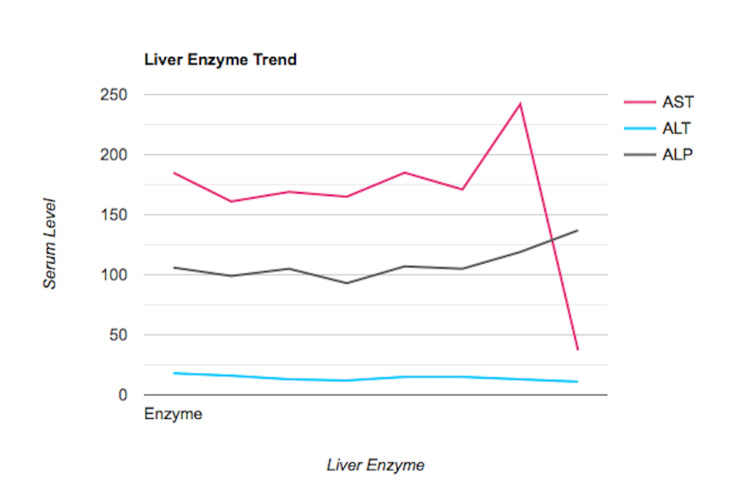
Graph illustrating liver enzyme trend AST = aspartate aminotransferase; ALT = alanine aminotransferase; ALP = alkaline phosphatase

During the initial consultation, she reported a nonspecific, mild, right-sided abdominal pain without prandial association or other exacerbating factors. She denied the use of alcohol, tobacco, or illicit drugs. She reported having had a cholecystectomy several years prior that was complicated by a bile leak and required endoscopic retrograde cholangiopancreatography-guided (ERCP) stent placement. Laboratory analysis revealed that the patient had evidence of elevated AST levels for the last several years without any associated elevations in alanine aminotransferase (ALT), alkaline phosphatase, or bilirubin (Figure 1). Physical examination findings were remarkable for mild, diffuse tenderness to palpation of the abdomen. MRI of the abdomen revealed mild dilation of the intrahepatic ducts, as expected post-cholecystectomy, but without overt evidence of advanced fibrosis. Autoimmune serologies were ordered and were unremarkable (Table 1). Inflammatory markers were ordered and were also unremarkable (Table 2). Serologies for viral hepatitis were negative, including hepatitis A virus (HAV), hepatitis B virus (HBV), and hepatitis C virus (HCV) (Table 3).

**Table 1:** Autoimmune workup

Autoimmune Serology	
Anti-Nuclear Antibody Titer	1:40 (Ref Range 1:40-1:80 Low Antibody Level)
Alpha-1 Antitrypsin	139 (Ref Range 83-199 mg/dL)
Actin IgG Antibody	<20 (Ref Range <20 U)
Cyclic Citrullinated Peptide IgG Antibody	<16 (Ref Range <20 U)
Mitochondrial M2 Antibody	<16 (Ref Range <20 U)
Anti-Smith Antibody	<1 (Ref Range <1.0 AI)
Histone Antibody	<1 (Ref Range <1.0 U)
Anti-Ribonucleoprotein Antibody	<1 (Ref Range <1.0 AI)
Single-Stranded DNA IgG Antibody	<69 (Ref Range <230 U/mL)
Rheumatoid Factor	<14 (Ref Range <14 IU/mL)
Myeloperoxidase Antibody	<1 (Ref Range <1.0 AI)
Proteinase-3 Antibody	<1 (Ref Range <1.0 AI)
Sjogren Syndrome A/B Antibody	<1 (Ref Range <1.0 AI)
Tissue Transglutaminase IgG Antibody	1 (Ref Range <4.0 U/mL)
Aldolase	4.8 (Ref Range <8.1 U/L)

**Table 2:** Inflammatory workup

Inflammatory Serology	
Creatine Kinase	104 (Ref Range 29-143 U/L)
Ceruloplasmin	37 (Ref Range 18-53 mg/dL)
Copper	165 (Ref Range 70-175 mcg/dL)
Ferritin	102.8 (Ref Range 13-150 ng/mL)
Transferrin	257 (Ref Range 188-341 mg/dL)
Lactate Dehydrogenase	184 (Ref Range 135-214 IU/L)
Haptoglobin	189.9 (Ref Range 20-230 mg/dL)
Erythrocyte Sedimentation Rate	30 (Ref Range 0-30 mm/hour)
Immunoglobulin M	151 (Ref Range 48-271 mg/dL)
Immunoglobulin G	1002 (Ref Range 694-1618 mg/dL)
Immunoglobulin A	285 (Ref Range 81-463 mg/dL)

**Table 3:** Infectious workup

Infectious Serology	
Hepatitis A IgM Antibody	Negative
Hepatitis B Core Antibody	Negative
Hepatitis B Surface Antigen	Nonreactive
Hepatitis C Antibody	Negative

**Figure 2:**
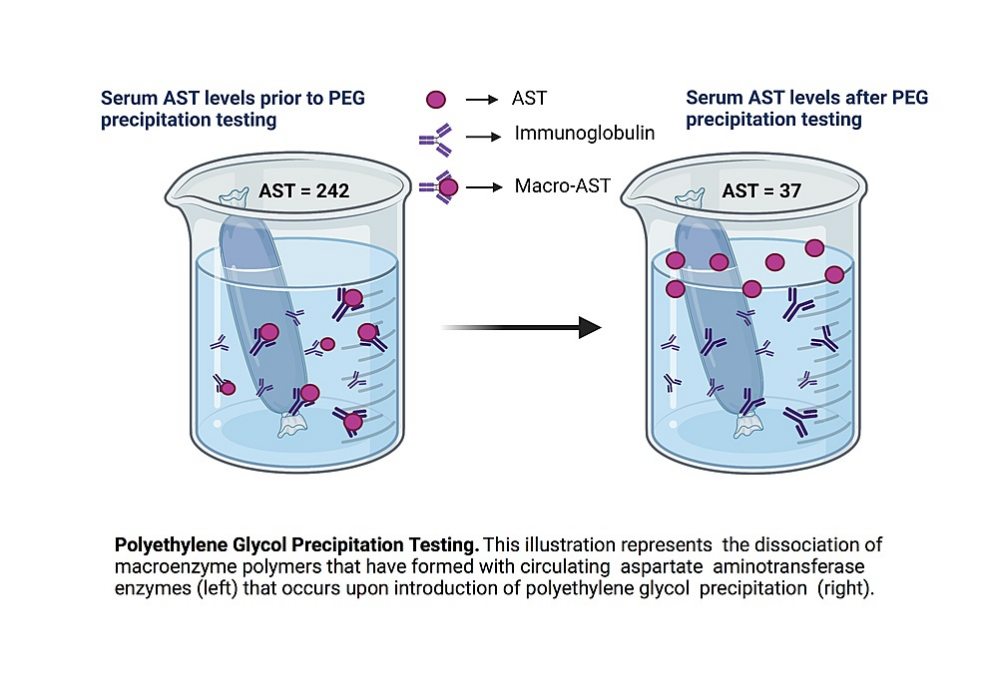
Illustration of polyethylene glycol precipitation testing AST = aspartate aminotransferase; PEG = polyethylene glycol; macro-AST = macroenzyme aspartate aminotransferase The image is created with BioRender.com and reprinted from "Dialysis" by BioRender on April 2021. [Retrieved from https://app.biorender.com/biorender-templates/figures/5c95180bc2753f33003dacd3/t-60c771c05da25200a7c5e48c-dialysis] Copyright 2021 by BioRender.

A specialized test for macro-AST syndrome was ordered. This involves precipitation of the blood specimen with polyethylene glycol (PEG). Upon dissolution in the PEG solution, AST multimers dissociate, allowing for a more accurate measurement of AST concentrations. Her test confirmed the presence of macro-AST; AST levels normalized from 242 to 37 (Figure 2). The patient was informed of her diagnosis and that this is a benign process with an excellent prognosis. She was discharged from the clinic, and no additional diagnostic tests or surveillance were performed.

## Discussion

There are two major theories to explain the underlying pathophysiology of macroenzyme formation. The first theory is the “antigen-driven theory” which postulates that a self-antigen is released from tissue which is then targeted and bound by a circulating antibody (i.e., molecular mimicry). The second is the “dysregulation of immune tolerance theory,” which postulates that macroenzymes form due to a dysregulated immune response similarly seen with autoimmune disorders [1-3,6,7]. The formation of macroenzymes appears to occur more often in the elderly (60 years and older) and appears to affect men and women equally [1,3].

Macroenzyme type I is formed by the binding of a serum enzyme with an immunoglobulin, most commonly IgG and IgA, creating a bulky immune complex that is difficult to clear through the kidneys [1,2]. The serum enzyme binds with the antigen-binding fragment (Fab) of the immunoglobulin creating a stable immune complex that is resistant to high temperatures and decreases the rate of clearance from the serum resulting in falsely elevated serum concentrations [1]. Clinically relevant type I macroenzymes include the following - alanine aminotransferase (ALT), aspartate aminotransferase (AST), amylase, alkaline phosphatase (ALP), creatine kinase (CK), lactate dehydrogenase (LDH), and lipase [1]. There are a few reports that indicate that these macroenzymes incidentally coexist in patients with underlying autoimmune diseases, which has sparked much debate on the role macroenzymes play in the pathogenicity of autoimmune disorders. There are three proposed mechanisms that could explain the role of macroenzymes in autoimmune disorders - (1) immune complex deposition, (2) interference with the native functioning of the antigen, and (3) direct cytotoxic effects on the tissues that express the antigen [1]. Regardless of this mechanistic relationship, there is insufficient data to support the correlation between macroenzymes and autoimmune disorders. Furthermore, the majority of reported cases of macroenzymes follow a benign clinical course.

Macroenzyme type II is formed through the binding of a serum enzyme with a foreign substance (i.e., medications) or through self-polymerization [1,2]. The following two mechanisms have been proposed in the formation of this type of macroenzyme - (1) bile solubilizes plasma membrane-bound enzymes in the setting of bile obstruction resulting in binding of the solubilized enzymes to hydrophobic lipoprotein carrier proteins in the serum subsequently forming a macroenzyme, and (2) in the setting of hepatic obstruction, the hepatobiliary plasma membrane breaks down releasing plasma membrane fragments bound to enzyme molecules into the circulation creating a macroenzyme [1]. Clinically relevant type II macroenzymes include the following - ALP, amylase, CK, gamma-glutamyltransferase (GGT), and leucine aminopeptidase [1]. Interestingly, the presence of macro-CK type II may have important clinical significance due to its reported association with severe liver disease and disseminated malignancy [1].

The detection of macroenzymes can be performed via ultracentrifugation, gel filtration chromatography (GFC), or polyethylene glycol (PEG) precipitation testing. The PEG precipitation test is the most cost-effective technique for screening suspected cases of macroenzymes in the serum. Precipitation in PEG removes the immunoglobulin component of the immune complex, allowing for dissociation of the enzymes and accurate measurement of their concentrations. There are limitations, including low sensitivity and the need for confirmatory testing [4,5]. In general, when a PEG precipitation test is performed the results are interpreted based upon the “PEG precipitable activity” (i.e., “% percent precipitable activity {PPA}”) and the reference range differs for each type of macroenzyme [4]. In particular, macro-AST has a reported % PPA reference range from 67.1% to 82.2% with any sample scoring above this indicating a high probability of macro-AST [4].

The differential diagnosis of elevated transaminases is broad and warrants further investigation to rule out any underlying intrinsic disease of the hepatobiliary system. Furthermore, it is essential to quantify the relative increases in AST and ALT as the ratio of AST:ALT can alter the most probable diagnoses and course of management [8]. Of note, AST is less specific for detection of liver damage in comparison to ALT, as it can also be found in striated muscle throughout the body. Acute myocardial infarction and rhabdomyolysis should be ruled out in the setting of elevated AST levels [9]. One of the most common causes of elevated liver enzymes in the United States is steatohepatitis. A substantial increase in AST in comparison to ALT should prompt investigation into alcoholic steatohepatitis, whereas an equivocal increase in both AST and ALT should prompt investigation into nonalcoholic steatohepatitis [8]. Significantly elevated liver enzymes should prompt investigation into potential toxic ingestions (aspirin and acetaminophen) or shock liver [10]. Shock liver should always be on the differential in a critically ill patient with sudden elevations in liver enzymes, especially ALT levels >10 times the upper limit of normal. Lastly, a full infectious workup to include a hepatitis panel and a thorough medication reconciliation is crucial in the investigation of elevations in liver enzymes.

The accurate and early detection of macroenzymes is clinically important as it can help to prevent unnecessary healthcare utilization and decrease the cost burden on the patient and the healthcare system. Many patients that are diagnosed with macro-AST go through years of unnecessary laboratory tests and imaging before arriving at the underlying diagnosis. The patient in this case underwent an extensive workup in relation to her isolated AST over the span of approximately five years before being correctly diagnosed with macro-AST. The laboratory analysis and testing that this patient underwent totaled approximately two thousand two hundred and fifty dollars, which is much higher than the eight dollars required for a PEG precipitation test. This leads to the most important lesson to be learned in this case, which is the proper utilization of healthcare resources when it comes to working up any isolated elevation in AST levels.

## Conclusions

The early detection and accurate diagnosis of macro-AST is important on both an individual and global healthcare scale. Although thoughtfully working up the many different etiologies of elevated transaminases is important, an isolated elevation in aspartate aminotransferase enzyme levels should prompt an investigation into other possible explanations to include macro-AST. The use of cost-effective tests such as the polyethylene glycol precipitation test allows for clinicians to quickly determine if an isolated elevation in serum enzymes is pathogenic or merely a benign entity masquerading as a clinically significant disease process.
